# Hydrothermal Synthesis of Silver Decorated Reduced Graphene Oxide (rGO) Nanoflakes with Effective Photocatalytic Activity for Wastewater Treatment

**DOI:** 10.1186/s11671-020-03323-y

**Published:** 2020-04-28

**Authors:** Muhammad Ikram, Ali Raza, Muhammad Imran, Anwar Ul-Hamid, Atif Shahbaz, Salamat Ali

**Affiliations:** 1 Solar Cell Applications Research Lab, Department of Physics, Government College University Lahore, Lahore, Punjab 54000 Pakistan; 2 Department of Physics, Riphah Institute of Computing and Applied Sciences (RICAS), Riphah International University, 14 Ali Road, Lahore, Pakistan; 3 State key Laboratory of Chemical Resource Engineering, Beijing Advanced Innovation Centre for Soft Matter Science and Engineering, Beijing Engineering Center for Hierarchical Catalysts, Beijing University of Chemical Technology, Beijing, 100029 China; 4 Center for Engineering Research, Research Institute, King Fahd University of Petroleum & Minerals, Dhahran, 31261 Saudi Arabia; 5 Department of Physics, Government College University Lahore, Lahore, Punjab 54000 Pakistan

**Keywords:** Silver, Nanoflake, rGO, Photocatalytic, Waste water

## Abstract

Graphene oxide (GO) was obtained through modified hummers method, and reduced graphene oxide (rGO) was acquired by employing heat treatment. Various concentrations (2.5, 5, 7.5, and 10 wt. %) of silver (Ag) were incorporated in GO nanosheets by adopting hydrothermal approach. Synthesized Ag decorated rGO photocatalyst Ag/rGO was characterized using X-ray diffraction (XRD) to determine phase purity and crystal structure. XRD patterns showed the formation of GO to Ag/rGO. Molecular vibration and functional groups were determined through Fourier Transform Infrared spectroscopy (FTIR). Optical properties and a decrease in bandgap with insertion of Ag were confirmed with UV-Visible (Uv-Vis) spectrophotometer and photoluminescence (PL). Electronic properties and disorders in carbon structures were investigated through Raman spectroscopy that revealed the existence of characteristic bands (D and G). Surface morphology of prepared samples was examined with field emission scanning electron microscope (FESEM). Homogeneous distribution, size, and spherical shape of Ag NPs over rGO sheets were further confirmed with the help of high-resolution transmission electron microscope (HR-TEM). Dye degradation of doped and undoped samples was examined through Uv-Vis spectra. Experimental results indicated that photocatalytic activity of Ag@rGO enhanced with increased doping ratio owing to diminished electron-hole pair recombination. Therefore, it is suggested that Ag@rGO can be used as a beneficial and superior photocatalyst to clean environment and wastewater.

## Introduction

Water on earth is akin to blood in our bodies. It is a key resource material for survival and development of all living species. Albeit 71% of the earth’s surface is covered with water, only 0.03% of total water is considered as freshwater that can be directly utilizable by humans through freshwater lakes, rivers, and shallow groundwater [1]. In recent decades, inadequate availability of clean drinking water has presented itself as an unrelenting global concern. Rapid growth of the world’s population and industrialization has led to increasing environmental pollution, such that around 750 million people face lack of access to clean water [2, 3]. Water reservoirs are recurrently contaminated by various hazardous pollutants containing heavy metal ions, dyes, oil, and other chemicals that are released from different leather tanneries and industries related to textile, rubber, paper, cosmetics, dyeing, plastic, and food [4]. According to the World Bank report, 17-20% of water pollution is instigated by textile industry. Annually ~ 1/10th million types of dyes are produced in numerous textile processes, among these dyes methylene blue (MB) 10-15% is directly released in the effluent. These pollutants create serious health issues such as cancer, skin irascibilities, allergy, and liver malfunctioning and are also harmful to aquatic life [4, 5].

To address these global problems, certain conventional treatment approaches such as ion exchange, electrolysis, carbon filter, chemical coagulation, biological methods, membrane filtration, and reverse osmosis (RO) are employed. Nevertheless, series of drawbacks and limitations are associated with these techniques including ineptness, complex procedure, high sludge formation, high implementation and operational cost, and use of large amounts of energy [4, 6, 7]. Thus, efficient technologies with aforementioned properties need to be developed; among such techniques, photocatalysis overcomes maximum deficiencies.

To date, photocatalytic degradation using inorganic semiconductor nanomaterials has exhibited vast desirability and interest for researchers owing to its excellent physical and chemical properties such as low toxicity, electrochemical stability, super oxidative capacity, cost-effectiveness, and environmental viability [2, 8, 9]. During the photocatalytic (PC) process, nanomaterials absorb greater visible light energy than bandgap initiated excitation between valence and conduction bands. Through charge separation, electron-hole pairs are generated. Free radicals (OH) oxidize organic compounds and degrade contaminants [8, 10].

On the other hand, some crucial factors are vital to determine PC performance, specifically surface area of photocatalyst since organic pollutants degrade primarily on the surface of semiconductor. Presence of robust light absorption capacity, fast interfacial redox rate, among various nanostructures; two-dimensional (2D) nanostructures tend to achieve these features more efficiently [11–14]. 2D nanomaterials also offer electron transportation channels due to reduced junctions and grain boundaries in contrast to other spherical nanocrystals. Quick transport of electrons diminishes the recombination rate and boosts PC degradation performance. So in this line, graphene oxide (GO) is a suitable candidate to endorse semiconductive PC efficiency [15–18].

In the last few decades, in addition to CNTs and other carbon-based nanomaterials, graphene with single atomic thick nanosheet emerged as an eye-catching candidate with a wide range of promising relevant properties including energy conversion, storage, and catalytic activities [19–21]. In studies regarding water treatment and distillation, owing to a large number of delocalized electrons conjugated in sp^2^ configuration of carbon network, graphitic carbon enriches the transportation of photo-electrons and significantly enhances photo-conversion efficiency of the system. Besides, GO exhibits a high absorption ability of organic materials in an aqueous medium [22, 23]. GO and reduced graphene oxides (rGO) afford PC reaction and, owing to their narrow bandgap, are promoted as visible light active semiconductor photocatalysts. Nevertheless, room for improvement is present as photo-conversion was found to be poor caused by the rapid recombination of electron-hole pairs on the surface.

Photo-conversion efficiency of photocatalysts based on GO/rGO can be enhanced by preventing electron-hole recombination. To achieve this aim, surface modifications were well developed with noble metal ions including platinum (Pt), palladium (Pd), silver, and gold nanoparticles (NPs). The silver among most studied noble metals is considered a likely candidate for modification of graphene and its analogs for PC relevance because of its low cost, matchless optical properties, higher chemical stability, and non-toxic nature. More immobility of silver nanoparticles decorated on rGO is acknowledged as enhanced performance, primarily due to increased reactive area and superior charge separation. Unique electron aggregation and transportation properties of GO through conjugated scheme drive hot electrons to reactive sites and suppress recombination [23]. Consequently, on behalf of aforesaid benefits, we aimed to synthesize different ratio of Ag content (2.5, 5, 7.5, 10) weight % with rGO photocatalyst through hydrothermal route to examined the photocatalyst efficiency and also prepared samples would characterized through several techniques to study structural optical and electronic properties.

## Methods

The current study was aimed to synthesize various concentrations of Ag into rGO nanosheets through hydrothermal route to investigate the photocatalyst efficiency.

### Materials

Graphite (99 %) and sodium nitrate (NaNO_3_) 99.9% were procured from “Sigma-Aldrich,” while sulfuric acid (H_2_SO_4_, 37%) and phosphoric acid (H_3_PO_4_) were acquired from “Analar.” Silver (Ag, 99.8 %), potassium permanganate (KMnO_4_, 99 %), and hydrochloric acid (HCL) were attained from “Merck.” All chemicals were used without further purification.

### Synthesis of GO

**Fig. 1:**
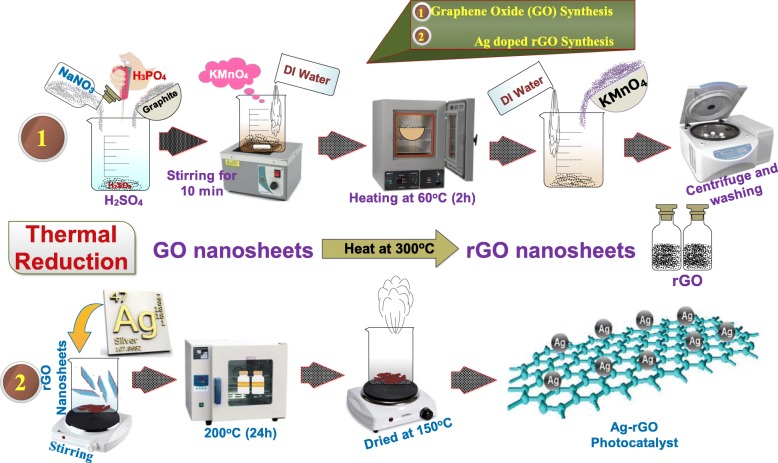
Synthesis process for GO and Ag-doped rGO

Modified hummers method was adopted to obtain GO. Graphite (5 g) and NaNO_3_ (2.5 g) were mixed in H_2_SO_4_ (108 ml) with 12 ml H_3_PO_4_. Mixture was magnetically stirred in an ice bath for 10 mins; further filtrate solution was dried in a muffle furnace at 60 °C for 2 h to eliminate moisture. Later, KMnO_4_ (15 g) was added slowly at maintained temperature to below 5 °C. Suspension was transferred to an ice bath for 2 h after vigorous stirring at 98 °C for 60 min while water was added continuously. Further deionized water was added until suspension volume was 400 ml after 5 mint H_2_O_2_ (12 ml) was mixed. Finally, the suspension was centrifuged and washed repeatedly with water and HCL product was dried at 60 °C and the pH of GO was found to be 5.7 after washing as illustrated in Fig. 1 [24, 25].

### Synthesis of Ag/rGO

The rGO was extracted from GO by thermal reduction GO has been reduced by keeping it at reduction temperature (300 °C) a sudden change in temperature causes elimination of functional groups and oxygen atoms from carbon planes and exfoliation of GO takes place to produce rGO [26]. The rGO can be considered as chemically-derived graphene, whose structure varied from one layer to multilayers [27]. Ag-doped rGO with various concentration ratios was synthesized hydrothermally, using 800 mg GO nanosheets incorporated with (25, 50, 75, and 100 mg) Ag in 80 ml deionized water under vigorously stirring for 20 min. The solution was then centrifuged (30 min) and subsequently transferred to a 100 ml Teflon-lined autoclave, sealed, and heated up at 200 °C (24 h). The final product was dried at ~ 200 °C as shown in Fig. 1 [9].

### Photocatalytic Activity

**Fig. 2:**
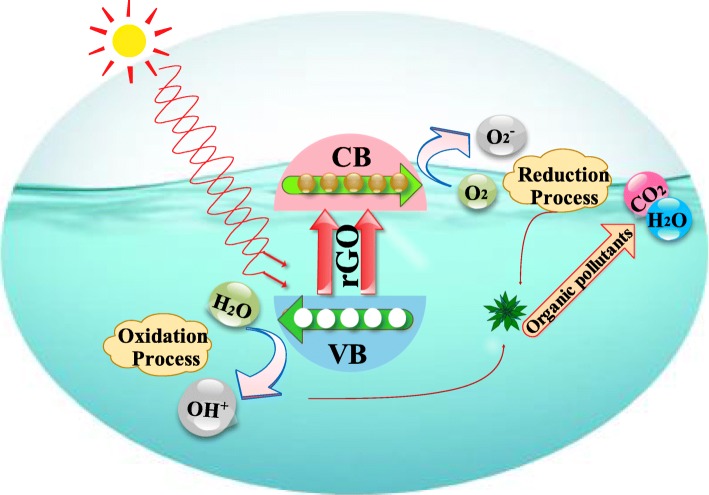
Photocatalytic mechanism for dye degradation in the presence of Ag/rGO

Photocatalytic activity of prepared products was evaluated by the degradation of synthetic methylene blue (MB) in aqueous medium as shown in Fig. 2. Dye (5 mg/500 ml) was prepared with 10 mg suspension of photocatalyst (0.025:1, 0.050:1, 0.075:1, and 0.1:1) under stirring (5 min) and exposed to dark for 30 min to achieve significant absorbance. A 60 ml of prepared solution with vigorous stirring was transferred to photo-reactor under mercury lamp (400 W and 400-700 nm) used as a visible light source. After light exposure for specified time intervals (20 min), suspension (3 ml) was collected to determine dye degradation. The concentration/absorbance of MB was examined with UV-Vis spectrometer; decolorization efficiency of the prepared photocatalyst was evaluated as:

1 $$ \mathrm{Degradation}\ \left(\%\right)=\left[1-\left(C/{C}_o\right)\right]\times 100 $$

where *C*_o_ is the absorbance at *t* = 0 and *C* is the absorbance at time *t* (specific time interval) [8, 10].

The mechanism of photocatalytic degradation of organic molecules is elucidated as follows (Fig. 2). When photocatalyst (Ag/rGO) is irradiated with photons of energy equal to or more than the bandgap energy of PC, then electrons (e^−^) are excited from the valence band (VB)

2 $$ \mathrm{PC}+ hv\to {e}^{-}\left(\mathrm{CB}\right)+{h}^{+}\left(\mathrm{VB}\right) $$

Generated electrons through irradiation can be readily trapped by O_2_ absorbed molecule on the surface of photocatalyst (PC) or dissolved O_2_ to give superoxide radicals, i.e., O_2_^•−^

3 $$ {e}^{-}+{\mathrm{O}}_2\to {\mathrm{O}}_2^{\bullet -} $$

Thus, O_2_^•−^ can react with H_2_O to produce hydroperoxy radical (H_2_O^•^) and hydroxyl radical (OH^•^), which are influential oxidizing agents that decompose organic molecules:

4 $$ {\mathrm{O}}_2^{\bullet -}+{\mathrm{H}}_2\mathrm{O}\to {\mathrm{H}\mathrm{O}}_2^{\bullet }+\mathrm{O} $$

Concurrently, photogenerated holes could be trapped by surface hydroxyl groups (H_2_O) on the surface of a photocatalyst to produce hydroxyl radicals (OH^•^):

5 $$ {h}^{+}+{\mathrm{OH}}^{-}\to {}^{\bullet}\mathrm{O}{\mathrm{H}}_{\mathrm{ad}} $$

6 $$ {h}^{+}+{\mathrm{H}}_2\mathrm{O}\to {}^{\bullet}\mathrm{O}\mathrm{H}+{\mathrm{H}}^{+} $$

Eventually, organic molecules will be oxidized to yield CO_2_ and H_2_O as follows:

OH + organic molecules + O_2_ → products (CO_2_ and H_2_O) (7)

Temporarily, slight recombination of positive hole and electron could take place which could reduce photocatalytic activity of prepared nanocatalyst [28].

### Materials Characterization

Crystal structure and phase-information of GO and Ag@rGO were investigated through XRD, by spectrum Bruker system (XRD, D2 Phaser, USA) equipped with monochromatized Cu K radiation of an average wavelength of 0.154 nm (5-80°) using a scan rate of 0.05/min. FTIR Perklin Elmer 3100 spectrometer with a spectral range of 4000–400 cm^−1^ with an increase of 32 scans and a resolution of 0.2 cm^−1^ was employed to detect functional groups and other molecular vibrations of prepared samples. Optical properties were recorded through UV-Vis spectrophotometer (TECAN infinite M200PRO) in the range of 200–700 nm. Surface morphology and interlayer distance of synthesized samples were observed using field emission scanning electron microscope (FESEM), JSM-6460LV and high-resolution transmission electron microscope (HR-TEM) Philips CM30, and JEOL JEM 2100F. To confirm GO, Ag/rGO flakes and vibration modes, Raman spectra were employed on Renishaw in through reflex confocal Raman microscope with a wavelength of 532 nm (6 mW) laser. Photoluminescence spectra of as-prepared and doped samples were recorded through spectrofluorometer (JASCO, FP -8300).

## Results and Discussion

**Fig. 3:**
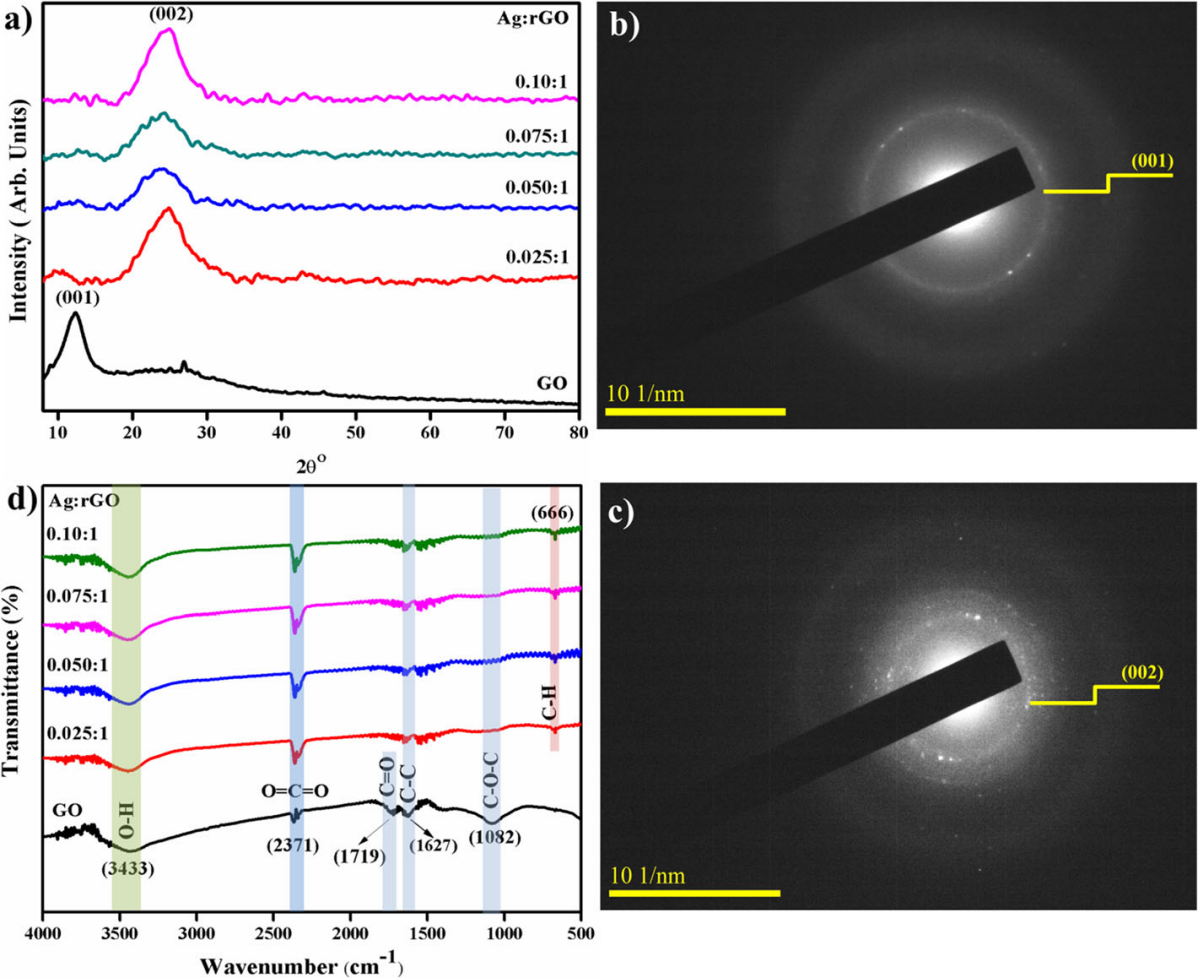
**a** XRD pattern. **b**, **c** SAED rings of as-prepared and Ag-doped RGO (**b**) 0:1 (**c**) 0.010:1. **d** FTIR spectra

The phase structure and crystallite size of prepared Ag inserted rGO nanosheets were examined using XRD analysis (Fig. 3a). Diffractogram of GO shows intense reflection located at ~ 10.27° attributed to (001) plane with an interlayer spacing of 0.80 nm [23, 29, 30]. Upon Ag doping broad peak originate at ~ 25.4°, which is recognized as the characteristic peak of graphene indexed as (002) plane (JCPDS No# 04-0783) of hexagonal graphite, with d spacing of 0.34 nm [23, 30–32]. Peak (001) reveals the graphite powder completely oxidized into GO and (002) peak endorsed the removal of polyhydrocarbon template in between two layers of rGO [30]. After Ag substitution, GO peak (001) shifted to a higher value to 2*θ* at 25.4° with lower d-spacing evident to redox reaction between graphene oxide and silver ions (Ag-rGO) and d-shifting value after reduction caused by the removal of oxygen-containing groups that intercalate between layers of reduced graphene oxide (rGO) as visible in XRD diffractogram [29, 32]. Average crystallite size assessed by Scherer’s equation:

8 $$ \mathrm{D}=\frac{\mathrm{k}\lambda }{\beta \cos \uptheta} $$

The crystallite sizes are found to be ~ 4.85, 11.3, 11.53, 11.6, and 28.3 nm respectively. In Eq. (8) *k* = 0.89, *β* = FWHM, *λ* = 0.154 nm, and *θ* = diffraction angle. Selected area electron diffraction (SAED) in Fig. 3b and c corresponding to XRD patterns of prepared samples exhibits distinct ring features and indicating hexagonal phase of GO and Ag/rGO manifested to well-crystallized products; also, ring indexing was consistent with XRD patterns.

Fourier transform infrared (FTIR) spectra of GO and Ag-doped rGO are illustrated in Fig. 3d. Observed peak ~ 3433 cm^–1^ corresponds to O-H stretching vibration [23]. Low transmittance peaks at 1719 cm^–1^assign to C = O stretching vibrations caused by COOH groups and the band at 1627 cm^−1^ assigned to the aromatic C-C stretching [10, 33]. Peak at ~ 2371 cm^−1^ assigned to COO groups [34]. Transmittance peak (~ 650 cm^−1^) is a fingerprint region of hybridized sp^2^ carbon bonding allotted as C-H bending vibration [35]. Band ~ 1082 cm^–1^ corresponds to C–O–C from hydroxyl stretching vibrations, upon doping, the peak value of functional groups on the doped sample slightly changed while their shapes remain similar [23, 29, 36].

**Fig. 4:**
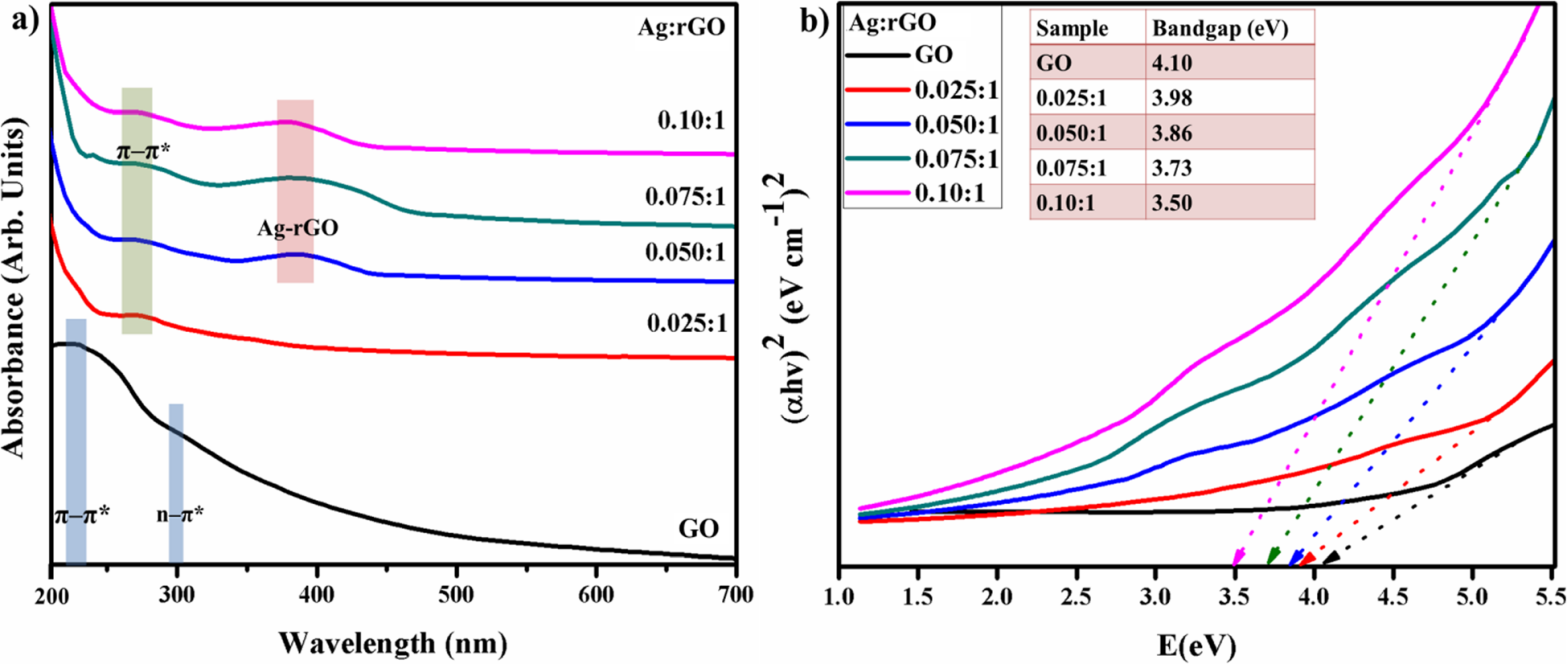
**a** UV-Vis spectra of GO and Ag-rGO. **b** Bandgap comparison

Optical properties in terms of absorbance and bandgap analysis of Ag-rGO photocatalyst were scrutinized through Uv-Vis spectrograph ranging from 200-700 nm as shown in Fig. 4a. The Uv-Vis spectrum of GO exhibits characteristics peak around 230 nm owing to *π*–π* transition of aromatic C–C bonds indicated restoring of an extensive conjugated framework of sp^2^ carbon atoms. Another shoulder peak observed at 300 nm attributed to *n*–π* transitions of C=O bonds [19, 23, 31]. Conversely, these two peaks became weaker in case of Ag/rGO corresponding to *π*–π* transition of aromatic C–C bonding found to be red-shifted at 270 nm that confirms the reduction of GO and indicate no restoring of electron conjugation of graphene [23, 29]. The absorption in the visible region (~ 400 nm) owing to their surface plasmonic resonance of Ag NPs that is further evidence to as visible light active photocatalyst for removal of organic bodies [23, 29, 37]. Bandgap was calculated by Tauc equation; *αhν* = *D*(*hν* − *Eg*)^*n*^ by plotting of (*αhν*)^2^ vs *hν* by extrapolated of linear fits, the band was calculated to be 4.10 eV for GO and 3.98 to 3.50 eV for Ag/rGO, bandgap gradually decreased with higher doping of Ag NPs clearly observed in Fig. 4b [38].

**Fig. 5:**
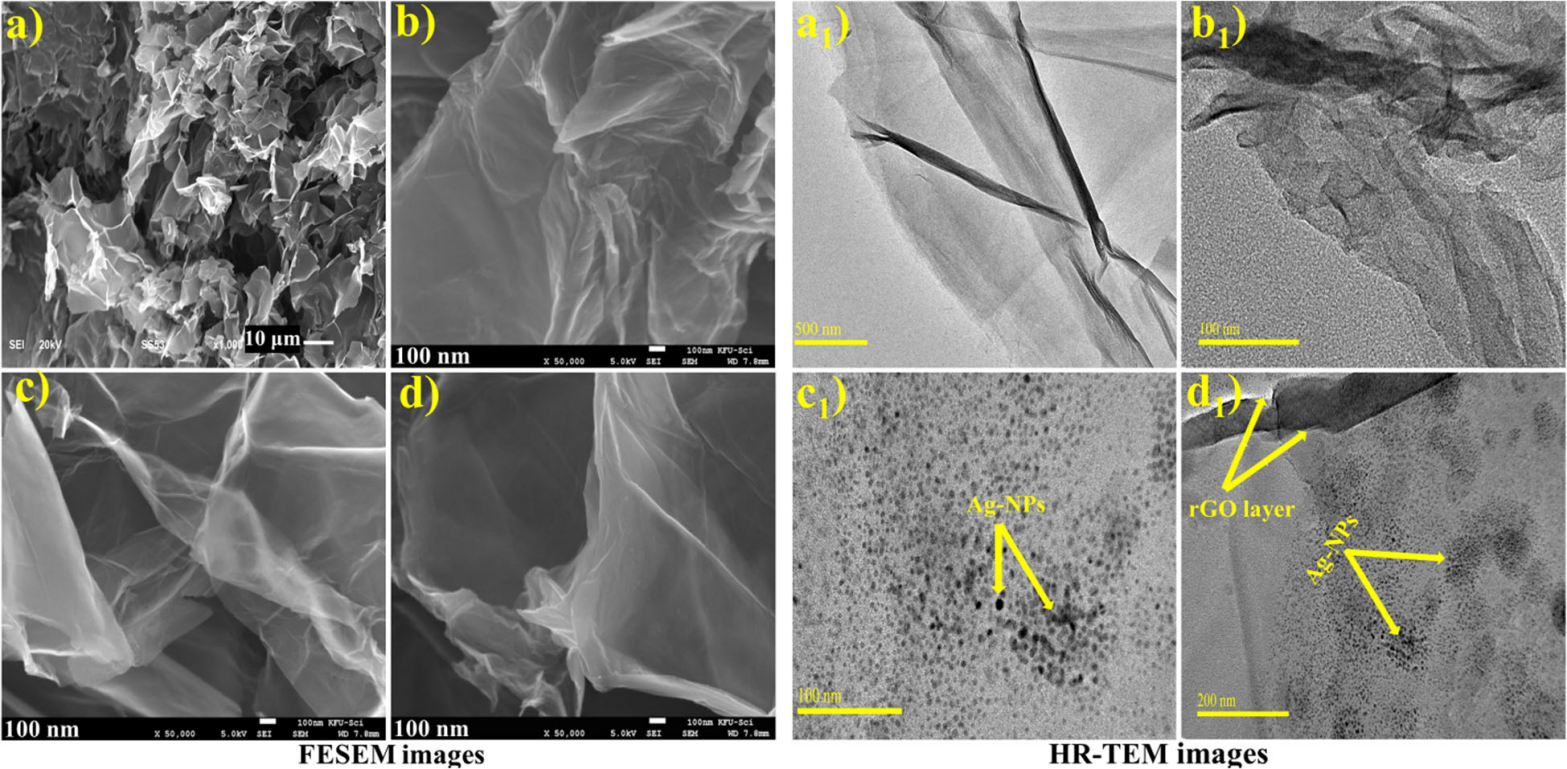
(**a**-**d**, **a**_1_-**d**_1_) FESEM and HR-TEM images of GO and Ag/rGO (**a**, **a**_1_) GO (**b**, **b**_1_) 0.050:1 (**c**, **c**_1_) 0.075:1 and (**d**, **d**_1_) 0.10:1

**Fig. 6:**
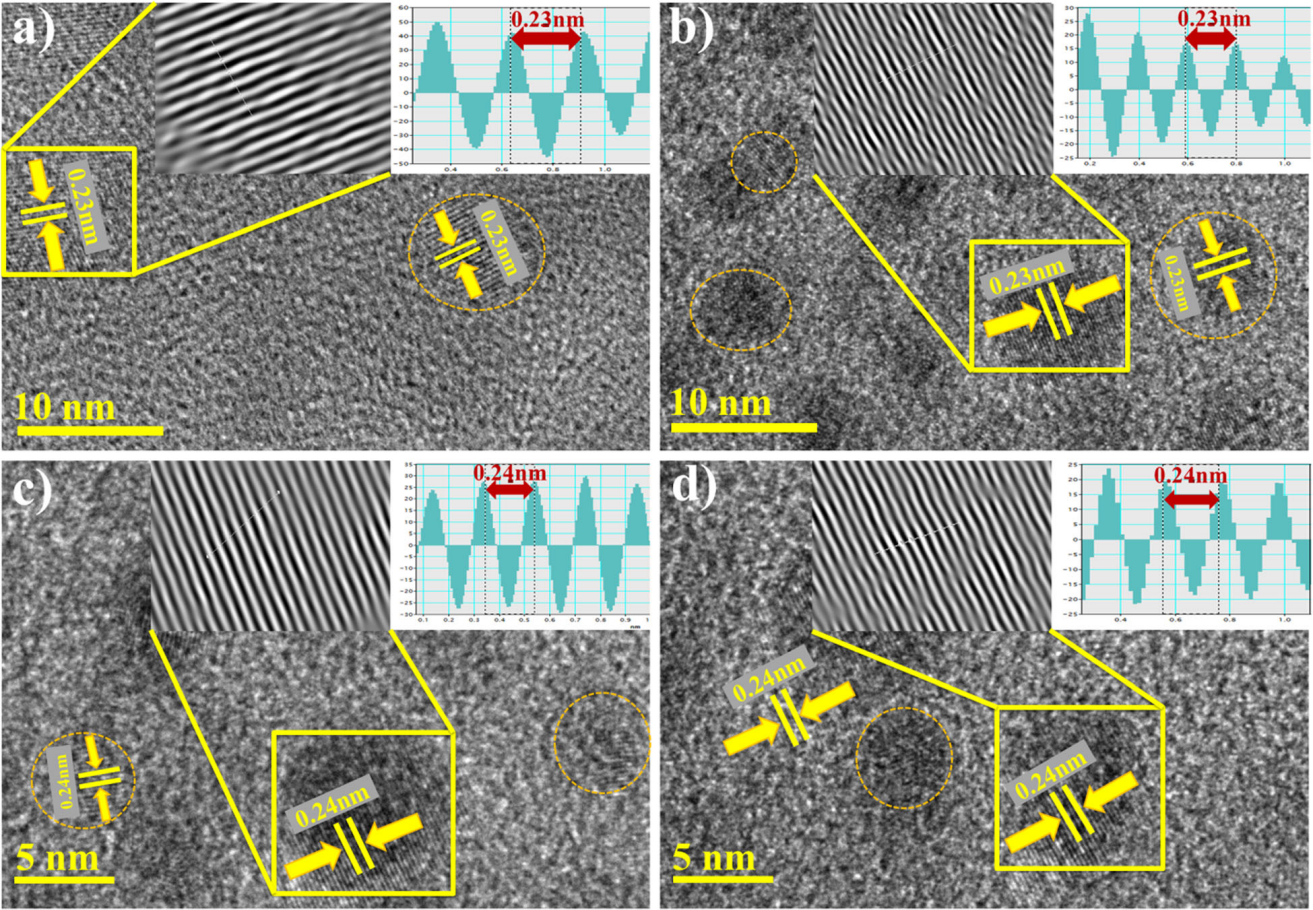
**a**-**d** d-spacing from HR-TEM images of Ag-rGO (**a**) 0.025:1 (**b**) 0.050:1 (**c**) 0.075:1 (**d**) 0.10:1

The morphological characteristics of GO and Ag-rGO samples elucidated through FESEM and HR-TEM showing in Fig. 5. GO images (Fig. 5a) show few layers of microstructures with rich wrinkles and fluffy morphology resembling with thin curtain. Images of Ag@rGO (Fig. 5b-d) show partially folded and curling transparent nanosheets with small fluctuations which are essential to endure thermodynamic stability of graphene, owing to its 2D crystal structure. Nanosheets exhibit extremely clean, silky, and wavy structures and this feature may be important to avoid aggregation of rGO sheets and maintain surface to facilitate attachment of Ag NPs on graphene sheets that can be visualized in HR-TEM images [36]. The corresponding HR-TEM images (Fig. 6 a_1_-d_1_), GO exhibits lamellar and sheet-like structure with a clean surface area (Fig. 6a_1_), in Ag-rGO sample (Fig. 5 b_1_) few stacking folds owing to their distortions from a high fraction of sp^3^ C–O bonds [29]. With increasing concentration of Ag NPs (Fig. 6 c_1_, d_1_) images revealed a well-dispersed and homogeneous scattering of spherical-shaped Ag NPs on the surface of rGO sheets with an average particle size of 10-12 nm [23, 29]. In Fig. 6 d_1_ with a higher concentration (10%) of Ag, aggregation of particles increased which is evident to doped species.

The extremely high resolution images up to 5 and 10 nm d-spacing of Ag/rGO samples can be clearly observed in Fig. 6a-d. Circled areas indicate the presence of Ag NPs with lattice spacing of Ag nanocrystal being approximately 0.235 nm [23, 29].

**Fig. 7:**
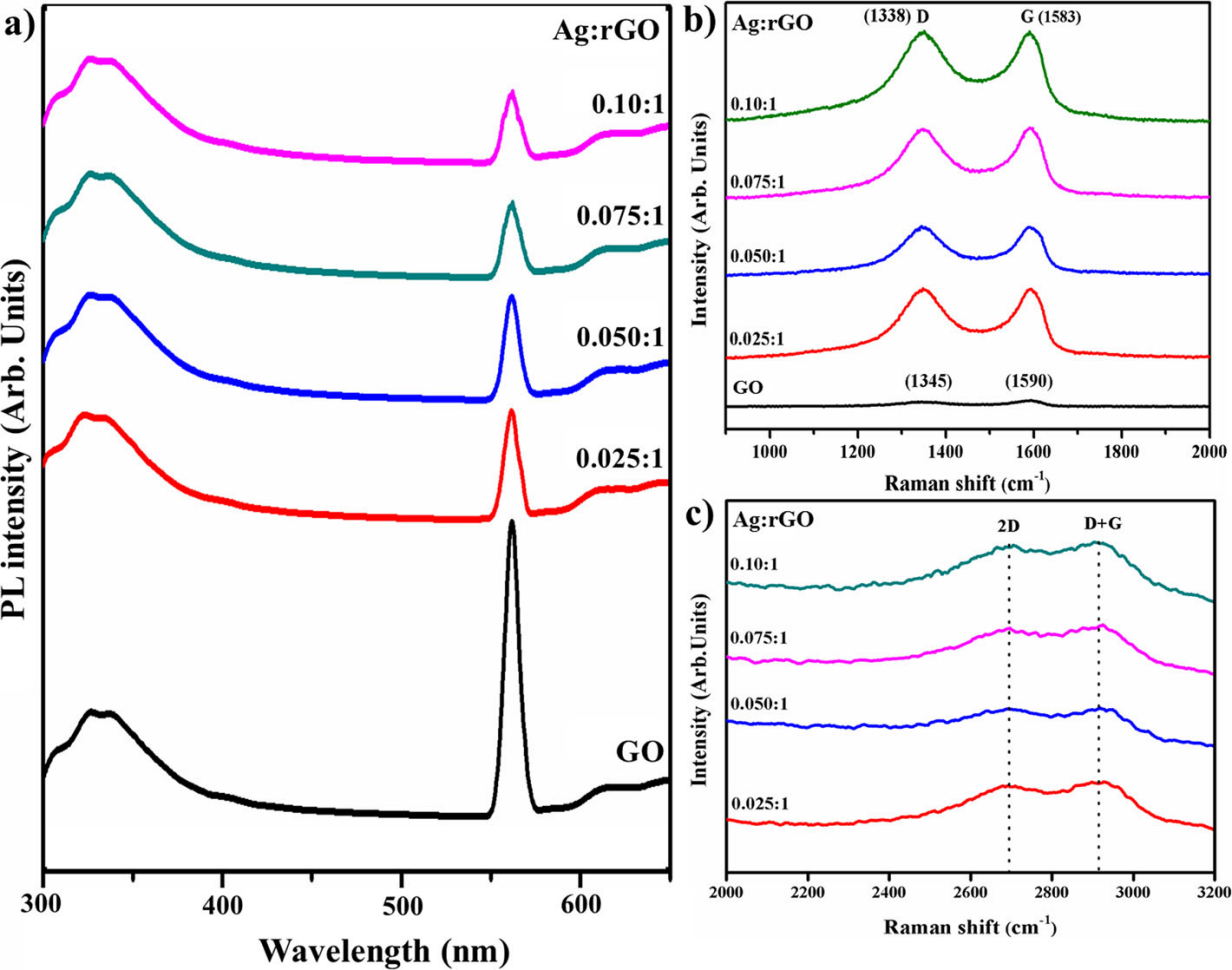
**a** PL spectra. **b** Raman spectra of prepared samples. **c** Zoom area of Raman spectra

Photoluminescence (PL) analysis was conducted to investigate lifetime, transfer, and trapping of electron-hole pair and study of the interaction between graphene nanostructures, its influence on photocatalytic response illustrated in Fig. 7a [39, 40]. Graphite exhibits no luminescence properties due to zero bandgap. Nevertheless, upon a decrease in size up to nano-scale, bandgap becomes wide caused by quantum confinement effect. In nanosheets of GO and rGO, oxides groups and carbon vacancies altered the graphene to form any carbon nano-cluster that demonstrates semiconductive behavior and luminescence phenomenon which can be influenced by size or fraction of chains and clusters [40, 41]. In PL spectra, luminescence peaks were located at ~ 330, 565, and 608 nm which is ascribed to electron-hole pair recombination in local state of sp^2^ carbon clusters incorporated with sp^3^ matrix. Therefore, rGO luminescence is due to disappeared oxygen functional groups that facilitate the percolation of pathways between sp^2^ clusters [40]. Significant peak at ~ 565 nm sharply decreased in case of rGO with reduction of GO oxide functional groups that are decreased and sp^2^ carbon clusters are expanded simultaneously [41].

Raman spectroscopy was deployed to probe electronic and structural properties of the control sample and Ag@rGO for distinguishing ordered and disordered carbon structures as demonstrated in Fig. 7b, c. In case of GO, two bands are located at ~ 1340 and ~ 1590 cm^−1^ assigned as D and G band, respectively. The D band is assigned to breathing mode of k point phonons with A_1g_ symmetry and band from sp^3^ carbon atoms; G band suggests a characteristic peak of sp^2^ hybrid structure which reveals symmetry and crystallizability of carbon and introduces E_2g_ phonon scattering of carbon atoms [32, 33, 36]. Moreover, D band is evident to surface defects and structural imperfections arise with attached hydroxyl and epoxide functions groups with carbon basal planes [36]. The G band is only Raman mode in graphene originating from a conventional first-order Raman scattering process and corresponds to in-plane zone center, doubly degenerate phonon mode (transverse (TO) and longitudinal (LO) optical) with E_2g_ symmetry [42]. In case of Ag-rGO Raman spectrum observed at 1338 cm^−1^ (D band), 1583 cm^−1^ (G band) and 2682 cm^−1^ (2D band) there is an additional peak centered at 2900 cm^−1^ (D + G band) that represents disorder due to combination scattering in Fig. 7b, c [31, 35, 42–45]. The D and 2D modes originate from the second-order double resonant process between non-equivalent k points in Brillouin zone (BZ) of graphene, as 2D band indicates second order of D band which alludes to overtone of D band with its existence owing two phone lattice vibrational processes; nevertheless, it is not associated with defects like D band in Fig. 7c [35, 41]. The variations in relative intensities of G and D band in Raman spectra of GO during reduction are usually designated to a change in electronic conjugation state. This change suggests an increase in the number of sp^2^ atomic domains following the reduction of GO [46]. The intensity ratio of D to G band defines disorder degree in graphite layers; I_D_/I_G_ = 0.87 for a doped free sample (GO), I_D_/I_G_ = 1.15 for Ag-doped samples and increase in ratio indicates a decrease in the average size of sp^2^ carbon domains after synthesis of Ag@rGO, while intensity ratio between 2D and G band (I_2D_/I_G_) which is 1.69, have been used to probe electrons concentration in rGO [31, 32, 35, 47].

**Fig. 8:**
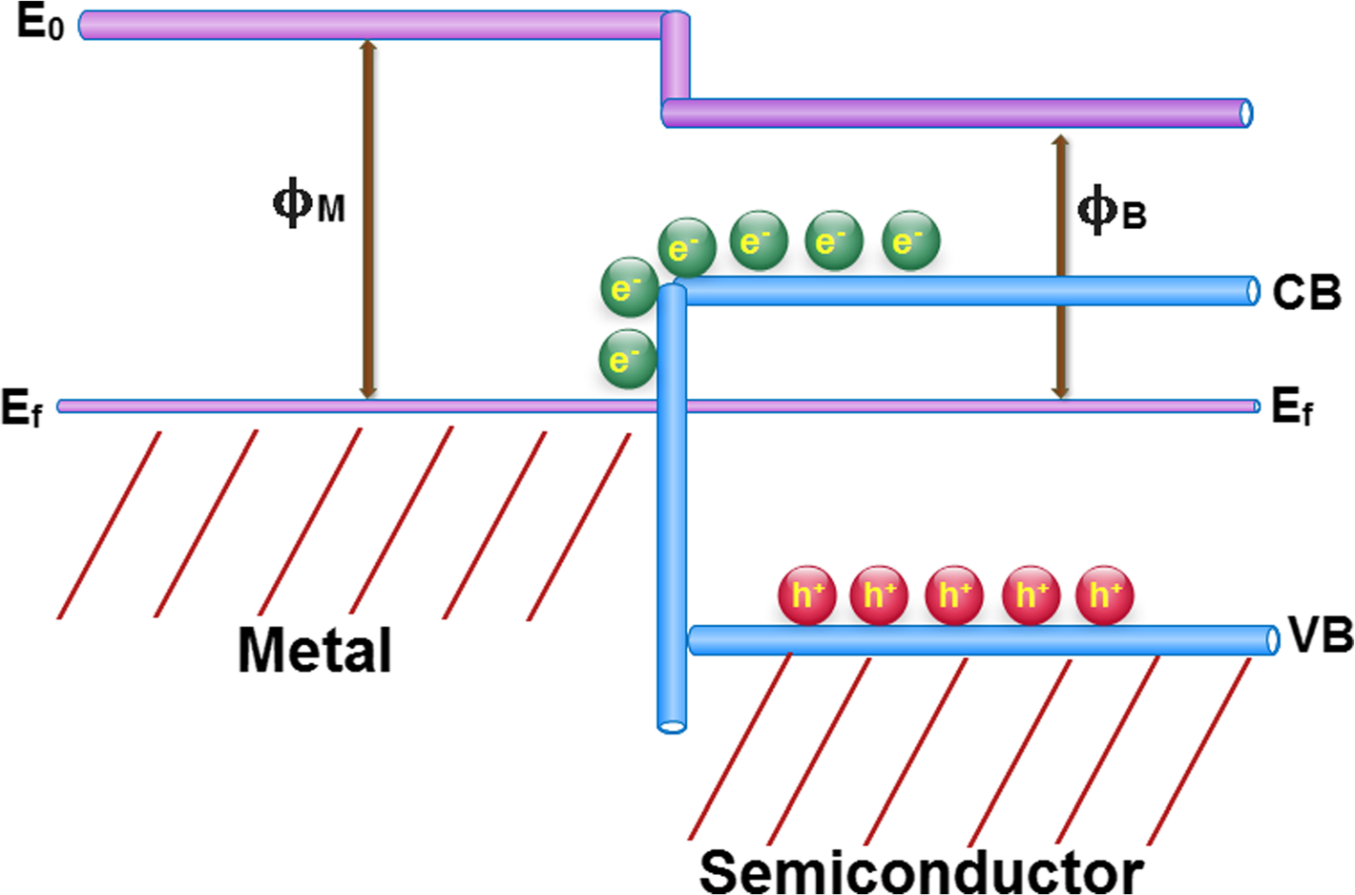
Qualitative electronic band structure of Ag decorated nanosheets

Ag nanoparticles, when doped in a semiconductor material, generate a contact potential difference due to their different work functions. This potential difference is called the Schottky barrier. As shown in Fig. 8, the band bending when a contact is formed after reaching equilibrium is dependent on the relative energies of the work functions of metal (ϕ_M_) and the semiconducting (ϕ_B_) components. This phenomenon can greatly enhance the charge separation efficiency, once it can induce the directional migration of photogenerated electrons from the semiconductor to the metal. In other words, it can lead to the generation of effective electron trapping site to suppress the electron-hole recombination [48].

**Fig. 9:**
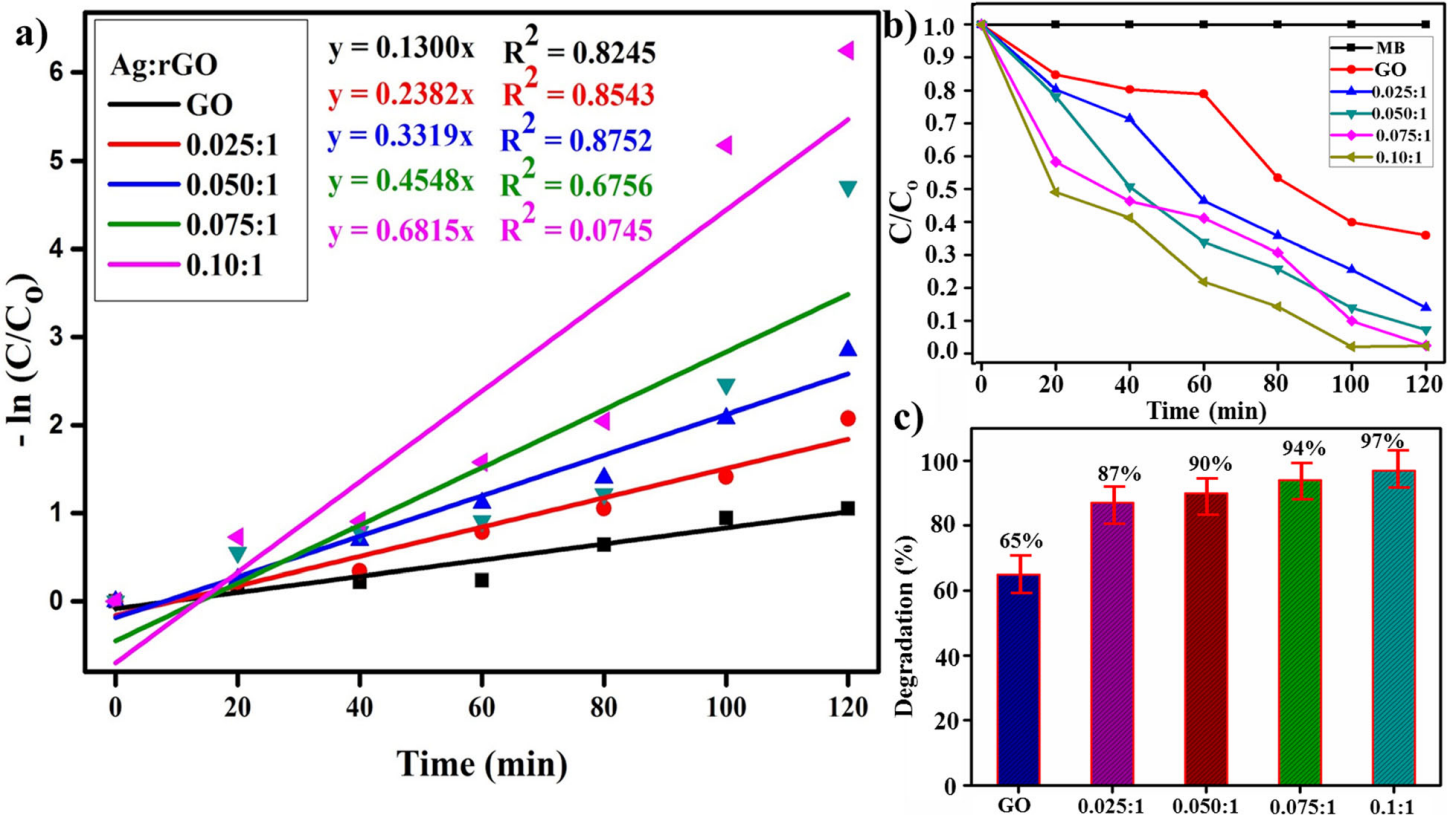
**a** Plot of -ln (Ct/C0) versus time spectra for dye reduction. **b** Plot of concentration ratio (C/C_0_) versus time. **c** Degradation (%) comparison of all samples

The photocatalytic activity of GO and Ag/rGO nanosheets occurred due to their high surface area, and low band-gap energy. Thus Ag/rGO exhibits substantial improvement in photo-degradation of MB and the dye degrades completely (Fig. 9b) in 120 min. The pseudo-first-order equation can be employed to elaborate on photocatalytic efficiency (Fig. 8a) of GO and Ag/rGO samples explicitly, using the following expression.

9 $$ -\mathit{\ln}\left({C}_t/{C}_o\right)= kt $$

where Co is the initial concentration of dye and *C*_t_ is the concentration at time, *k* is the apparent rate constant of degradation process that shows in absorbance plot (Fig. 9a), i.e., value of *k* for GO is about 0.1300 min^−1^ and *k* extraordinarily increases in case of Ag/rGO (0.1300 min^−1^ to 0.7459 min^−1^). Figure 9c reveals compression of % degradation with time, GO shows 65% efficiency and a gradual increase with doping concentration. Ag/rGO (0.10:1) shows maximum % degradation up to 100% which is likely due to synergetic effects of Ag NPs [49, 50]. Finally, on the basis of these findings in the present study, it can be suggested that Ag/rGO is an excellent product that can be used for purification of water from organic dyes.

## Conclusion

GO was successfully obtained through the modified hummers method and rGO was synthesized from thermal treatment during the insertion of Ag (2.5, 5, 7.5, and 10 wt.%) via hydrothermal route. According to XRD pattern, peak shift and decrease in d-spacing (0.34 to 0.023 nm) point toward redox reaction of GO upon Ag-doping with hexagonal crystal structure; average crystallite size increase (4.85 to 15.6 nm) with substitution of Ag is observed. FTIR spectra confirmed transmittance peak around 650 cm^−1^ which is a fingerprint region of hybridized sp^2^ carbon bonding allotted as C-H bending vibration and reveals information about other attached functional groups. The characteristic peak attributed to *π*–π* and *n*–π* bonding and redshift in peaks. It endorses the presence of Ag as elucidated with UV-Vis spectroscopy, an obvious decrease in bandgap energy (4.10 to 3.50 eV) with increased doping ratio that was calculated with the help of Tauc equation. Morphological features show stacking layers of GO and Ag/rGO with a lattice spacing of ~ 0.235 nm, spherical shape, and size (10-12 nm) of Ag NPs visualized through HR-TEM. The carbon atoms of local state sp^2^ clusters incorporated with sp^3^ matrix, significant peak decrease in case of rGO and expanded sp^2^ carbon cluster upon doping was confirmed with PL spectra. A_1g_ symmetry in sp^3^ carbons atoms at D band, sp^2^ hybrid structure that reveals symmetry and crystallizability of carbon and introduces E2g phonon scattering of a carbon atom and surface defects were calculated through Raman spectra. Photocatalytic activity responds to Ag/rGO (0.10:1) and degrades 100% of MB concentration. These findings suggest that prepared nanocatalyst shows no hazard behavior in water treatment and is an excellent nanocatalyst for the elimination of organic pollutants from wastewater.

## Data Availability

All data are fully available without restriction.

## Abbreviations

UV-Vis: Ultra-violet visible spectroscopy
XRD: X-ray diffraction
FTIR: Fourier transform infrared spectroscopy
EDS: Energy dispersive X-ray spectroscopy
SEM: Scanning electron microscopy
TEM: Transmission electron microscopy
JCPDS: Joint committee on powder diffraction standards
